# Regulation of Plant Developmental Processes by a Novel Splicing Factor

**DOI:** 10.1371/journal.pone.0000471

**Published:** 2007-05-30

**Authors:** Gul Shad Ali, Saiprasad G. Palusa, Maxim Golovkin, Jayendra Prasad, James L. Manley, Anireddy S.N. Reddy

**Affiliations:** 1 Department of Biology and Program in Molecular Plant Biology, Colorado State University, Fort Collins, Colorado, United States of America; 2 Department of Biological Sciences, Columbia University, New York, New York, United States of America; University of Leeds, United Kingdom

## Abstract

Serine/arginine-rich (SR) proteins play important roles in constitutive and alternative splicing and other aspects of mRNA metabolism. We have previously isolated a unique plant SR protein (SR45) with atypical domain organization. However, the biological and molecular functions of this novel SR protein are not known. Here, we report biological and molecular functions of this protein. Using an in vitro splicing complementation assay, we showed that SR45 functions as an essential splicing factor. Furthermore, the alternative splicing pattern of transcripts of several other SR genes was altered in a mutant, *sr45-1*, suggesting that the observed phenotypic abnormalities in *sr45-1* are likely due to altered levels of SR protein isoforms, which in turn modulate splicing of other pre-mRNAs. *sr45-1* exhibited developmental abnormalities, including delayed flowering, narrow leaves and altered number of petals and stamens. The late flowering phenotype was observed under both long days and short days and was rescued by vernalization. *FLC*, a key flowering repressor, is up-regulated in *sr45-1* demonstrating that SR45 influences the autonomous flowering pathway. Changes in the alternative splicing of *SR* genes and the phenotypic defects in the mutant were rescued by *SR45* cDNA, further confirming that the observed defects in the mutant are due to the lack of SR45. These results indicate that SR45 is a novel plant-specific splicing factor that plays a crucial role in regulating developmental processes.

## Introduction

SR proteins constitute a highly conserved family of structurally and functionally related non-snRNP proteins with multiple roles in pre-mRNA splicing and other aspects of RNA metabolism [1]–[5]. These proteins have a modular domain structure with one or two N-terminal RNA recognition motifs (RRMs) and a C-terminal arginine/serine-rich (RS) domain. The RRM, which confers RNA-binding specificity, binds to specific regulatory sequences in pre-mRNA, and the RS domain mediates protein-protein and protein-RNA interactions in the splicing machinery [3], [6]. An important feature of SR proteins is that any one of them can complement splicing-deficient S100 extracts in splicing of pre-mRNA substrates with consensus splice sites [7]. Based on this property, SR proteins have been called essential or general splicing factors. SR proteins in animals function as essential splicing factors in constitutive pre-mRNA splicing and also regulate alternative splicing (AS) by influencing splice site selection in a concentration-dependent manner [3], [7]. During formation of the spliceosomal E-complex, ASF (alternative splicing factor)/SF2 (splicing factor 2), one of the SR proteins, recruits U1 snRNP to the 5′ splice site by interacting simultaneously with the pre-mRNA and the U1-70K protein [3], [6]. SR proteins (e.g., SC35 and ASF/SF2) are also involved in bridging 5′ and 3′ splice sites by interacting concurrently with U1-70K and U2AF^35^ [8]. Furthermore, SR proteins facilitate incorporation of the tri-snRNP complex (U4/U6.U5 snRNP) into the spliceosome and promote base pairing between U2 and U6 snRNA [3], [7]. Some animal SR proteins that shuttle between the nucleus and cytoplasm function in mRNA export, mRNA stability and/or translation [1]. These studies underscore the importance of this family of proteins in RNA metabolism. Although, SR proteins show functional redundancy in in vitro splicing assays, in vivo studies with non-plant systems indicate that some SR proteins are redundant whereas others are not [4].

In animals there are 11 SR splicing factors whereas this family of proteins is considerably expanded in plants with 19 in Arabidopsis [9]–[11] and 23 in rice [12], [13]. Part of this expansion is attributed to differences in splice site recognition between plants and animals [10]. In support of this notion, several SR proteins have been shown to interact with U1-70K, a U1 snRNP specific protein that plays important roles in constitutive and regulated splicing [1], [14]–[18]. Some plant SR proteins appear to be orthologs of metazoan SR proteins whereas others are unique to plants with novel structural features. Seven of the 19 Arabidopsis SR proteins have no counterparts in animal systems [10], [11].

**Figure 1:**
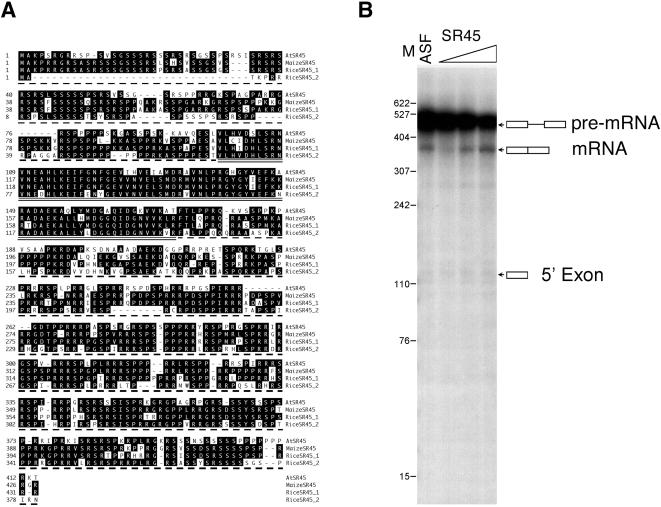
Arabidopsis SR45 is a splicing factor. (A) Alignment of SR45 amino acid sequences from Arabidopsis (AtSR45, At1g16610), rice (RiceSR45_1, Accession AK070420; RiceSR45_2, Accession AK063761) and maize (MaizeSR45, Accession BT016650). The RRM domain is underscored and the RS domains at the N-and C-terminus are indicated by dashed underlines. Identical amino acids are shown by reverse lettering. Dashes indicate gaps in alignment. (B) In vitro splicing of β-globin pre-mRNA in S100 cell extract supplemented with either 25 ng of ASF/SF2 (ASF) or increasing amounts (10, 30 and 90 ng) of purified SR45 (SR45) expressed in insect cells. The positions of pre-mRNA, spliced mRNA and 5′ exon are indicated to the right of the blot. Boxes and a line indicate exons and an intron, respectively.

SR45 was isolated as a U1-70K interacting protein in a yeast two-hybrid screen [17]. Unlike typical SR proteins, which contain a single RS domain at the C-terminus, SR45 has two distinct RS domains, one on either side of the RRM (Figure 1A). Using a GFP-SR45 fusion protein, we showed that SR45 localizes to nuclear speckles and its mobility is regulated by ATP and transcription [19], [20]. However, the molecular and biological functions of SR45 protein are not known. In this study, using an in vitro splicing complementation assay, we show that SR45 is an essential splicing factor. Further, the splicing patterns of several other SR genes is affected in a T-DNA insertion mutant, *sr45-1*, suggesting that, in addition to its role in constitutive splicing, it also plays a role in regulated splicing. Phenotypic analyses of *sr45-1* plants indicate an important role for SR45 in regulating multiple plant-specific developmental processes including plant size, flowering time and morphology of organs. The work reported here is significant because it shows not only that SR45 affects several developmental processes but also that a plant-specific SR protein with novel domain organization is a bonafide SR splicing factor.

## Results

### SR45 Is Present in Flowering Plants but Not in Algae and Animals

To identify Arabidopsis SR45 homologs in other organism we searched all the available plant, fungal and animal genomes and EST sequence databases (see Materials and Methods ). These searches revealed the presence of SR45 in rice, maize and in many other flowering plants, but not in algae (*Chlamydomonas reinhardtii*; *Cyanidioschyzon merolae*), a diatom (*Thalassiosira pseudonana*) and animals. These results indicate that SR45 has appeared later in evolution only in the flowering plants clade. Interestingly, there are two *SR45* genes in rice (accessions AK070420 and AK063761) as opposed to one in Arabidopsis, indicating that *SR45* may have undergone duplication after the divergence of monocots and dicots. A comparison of the amino acid sequences of Arabidopsis SR45 with homologs from rice and maize (accession BT016650) showed similar domain organization and considerable sequence conservation (51–80% similarity) (Figure 1A). The absence of SR45 in animals and algae and its presence in flowering plants suggest that SR45 may have evolved to perform functions that are specific to flowering plants.

### SR45 Protein Expressed in Insect Cells Complements Splicing-Deficient S100 Cell Extract

An important property of SR proteins is that each one is sufficient to complement splicing-deficient HeLa cell S100 extract in splicing of at least some pre-mRNA substrates. Because its domain structure is different from typical SR proteins, SR45 is not classified as an SR protein in a recent report [21]. Furthermore, animal proteins with similar domain organization such as human Tra2a and Trab cannot substitute for the essential splicing function of SR proteins [22]. To determine if SR45 functions as an essential splicing factor, we expressed SR45 as a His.tag fusion in insects cells and used the purified protein to analyze its splicing activity in S100 extract with a β-globin pre-mRNA substrate. Remarkably, the purified SR45 was able to activate splicing of β-globin pre-mRNA in a concentration-dependent manner and at a level comparable to recombinant human ASF/SF2 (Figure 1B). These results provide evidence that SR45, despite its unique domain organization, is a bonafide SR protein and functions as an essential splicing factor.

### Molecular Characterization of the *sr45-1* Mutant

**Figure 2:**
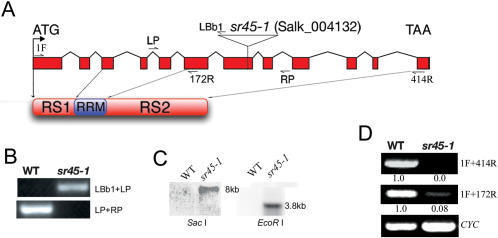
Molecular characterization of *sr45-1*plants. (A) Gene structure of *SR45* (At1g16610). Filled rectangles are exons; thin lines are introns; inverted open triangle indicates the position of the T-DNA insertion in the 7^th^ exon. The location of various primers used in PCR is indicated by half-head arrows. The schematic diagram below the gene structure shows domain organization of SR45 protein. The corresponding gene positions coding for the N-terminal arginine/serine rich (RS1) domain, the middle RNA recognition motif (RRM) and the C-terminal RS2 domain are indicated by downward arrows. (B) Verification of the *sr45-1* insertion in the genomic DNA by PCR with *SR45*-specific primers (LP and RP) and the T-DNA-specific primer LBb1; the locations of these primers relative to the T-DNA insertion are shown in (A). (C) Southern blot showing single insertion of T-DNA. Genomic DNA from WT and *sr45-1* was digested with either SacI or EcoRI and probed with a ^32^P-labeled T-DNA probe. (D) RT-PCR expression analyses of *SR45* in WT and *sr45-1* plants using *SR45*-specific primers (1F and 414R, which will amplify full-length *SR45*; 1F and 172R will amplify a truncated *SR45* transcript before the T-DNA insertion). Cyc, cyclophilin product was amplified to show equal amount of cDNA template in PCR. PCR product in WT and *sr45-1* was normalized to cyclophilin expression in WT and *sr45-1*, respectively. Numbers below each panel indicate the level of transcript in WT and *sr45-1* plants. The level of *SR45* transcript in WT is considered as 1.

To analyze the in vivo function of SR45, we characterized a T-DNA insertion mutant of *SR45* (Figure 2A). Genomic PCR analyses with *SR45*-specific primers (LP+RP) that flanked the T-DNA insertion site amplified the expected size *SR45* product from wild-type (WT) but not from the mutant indicating that *SR45* is disrupted (Figure 2B). In contrast PCR with the T-DNA-specific primer (LBb1) and *SR45*-specific primer (LP) amplified a product from the mutant but not from WT (Figure 2B), confirming that the insertion is in *SR45*. Insertion of T-DNA at a single locus was verified by Southern analysis (Figure 2C). RT-PCR with *SR45*-specific forward and reverse primers (1F+414R) amplified the expected size product from WT but not from the mutant (Figure 2D). To test if a truncated transcript upstream of the insertion is produced, PCR was performed with forward primer, 1F and a primer that is complementary to SR45 before the T-DNA (172R). The expected size product was amplified indicating that a truncated SR45 transcript is produced in the mutant (Figure 2D). However, the level of the truncated transcript is only 8% of the full-length transcript in WT, indicating that it may be unstable. This mutant was named *sr45-1* and was used in the experiments reported below.

### The Alternative Splicing Pattern of Several Related SR Genes is Altered in the *sr45-1* mutant

**Figure 3:**
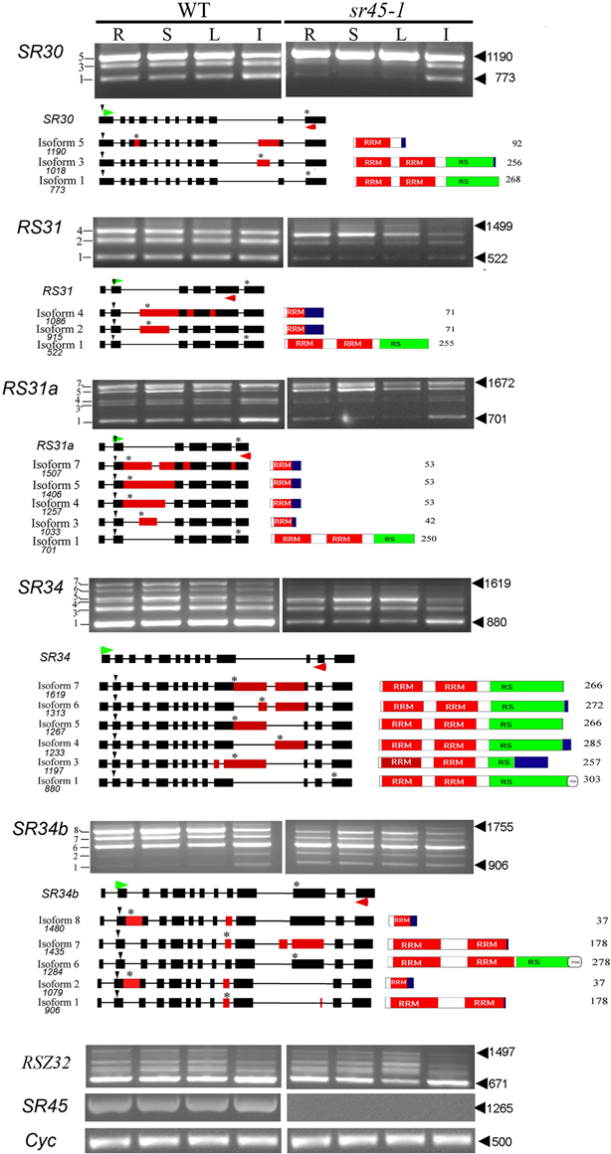
Expression and alternative splicing of pre-mRNAs of Arabidopsis *SR* genes in different organs is altered in the *sr45-1* plants. Expression levels were analyzed by RT-PCR with primers specific to each *SR* gene. Sequences of forward and reverse primers used are shown in Table S3. An equal amount of template in each reaction was verified by amplifying a constitutively expressed cyclophilin. The name of the *SR* gene is shown on the left of each panel. DNA sizes are indicated on the right. Isoform number is indicated on the left side of the gel. R, root; S, stem; L, leaf and I, inflorescence. Schematic diagrams in the bottom panel for each gene show the gene structure and its alternatively spliced mRNA isoforms (Numbers below each isoform indicate the number of nucleotides). Predicted proteins from splice variants are shown to the right of each isoform. Exons are filled rectangles and introns are thin lines. Black rectangles represent constitutively spliced exons whereas the red rectangles indicate the included regions in splice variants. Vertical arrowhead and ‘*’ show start and stop codons, respectively; Horizontal green and red arrowheads above and below gene structures indicate the position of forward and reverse primers, respectively. In the schematics of predicted proteins, numbers to the right are the number of amino acids in the protein. RRM, RNA recognition motif, RS, Arginine/Serine-rich domain. Blue rectangle indicates a stretch of amino acids that are not present in functional SR proteins.

To gain insight into the molecular mechanism of SR45, we analyzed the expression pattern of the alternative transcripts of all 19 SR genes in different organs in *sr45-1* and WT. Figure 3 shows that the AS patterns of *SR30, RS31, RS31a, SR34* and *SR34b* were different between *sr45-1* and WT. Sequencing of the alternative transcripts showed that, except for *SR34b*, the smallest transcript codes for a functional protein, whereas the rest code for truncated proteins (Figure 3). In the vegetative tissues of *sr45-1*, the amounts of *SR30* mRNA isoform 1 and 3 diminished to negligible levels, whereas the level of isoform 5 increased by approximately 2 fold (Figure 3, SR30). In inflorescence, isoform 1 and 3 showed reduced abundance relative to isoform 5. Similarly, in *sr45-1*, the smaller size transcripts of *RS31* (isoform 1 and 2) and *RS31a* (isoform 1, 3 and 4) were also diminished. In some cases, this reduction was accompanied by a moderate increase in the longer size products in some genes (e.g. *SR30*). In the case of *SR34*, there was an overall decrease in all transcripts in *sr45-1*. The abundance of the two smaller transcripts (isoform 1 and 3) in all tissues decreased. In stems and leaves, but not in roots and inflorescence, this reduction was accompanied by an increase in isoform 5. The abundance of the longest isoforms significantly diminished but this may have resulted due to an overall decrease in transcript level in *sr45-1* relative to WT. In all tissues, the abundance of the intermediate size transcript (isoform 6) of *SR34b*, which codes for full-length protein, remained similar between *sr45-1* and WT. In vegetative tissues, the abundance of the longer transcripts (isoform 7 and 8) decreased and the smaller size transcripts (isofrom 1 and 2) increased in *sr45-1*. In inflorescence of *sr45-1*, however, isoform 7 and 8 decreased but there was no significant change in the levels of isoform 1 and 2. The pattern of alternative transcripts of the rest of the SR genes, except for very subtle changes in a few transcripts, remained similar between *sr45-1* and WT (*RSZ32* and data not shown). Sequence analyses of the affected transcripts show that the longest intron is either partially retained or excluded in the alternative transcripts. Since mutation in *SR45* caused a decrease in the smaller size products and an increase in the longer size product, SR45 likely functions by influencing splicing of the longest intron to maintain a balance in the relative abundance of alternative transcripts. Overall these analyses indicate that SR45 affects the AS of a subset of transcripts of SR protein-encoding genes, which in turn may regulate the splicing of other transcripts and eventually control the different phenotypes observed in *sr45-1*plants.

### *sr45-1* Plants Show Delayed Flowering and Altered Leaf Morphology

**Figure 4:**
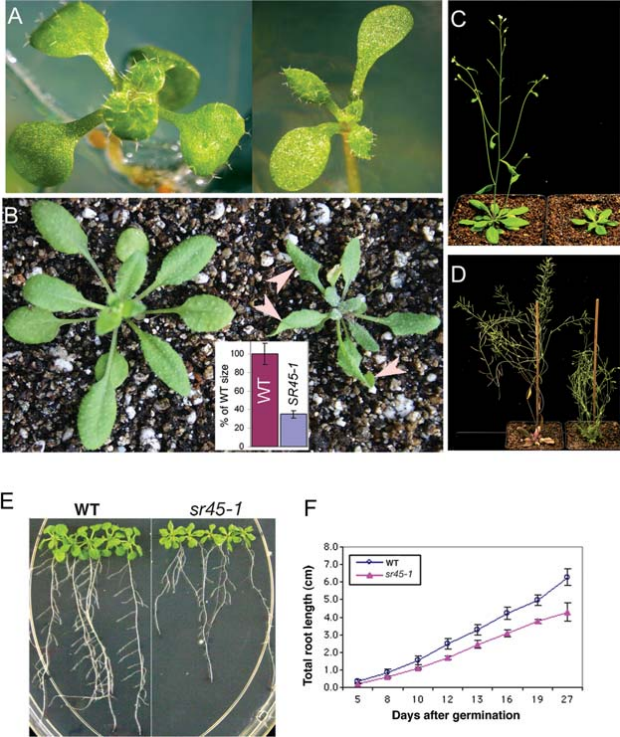
Phenotypic characterization of *sr45-1*plants. (A–D) Phenotypes of *sr45-1* at different growth stages grown under long-days (16-h photoperiod). WT plants are shown on the left side and *sr45-1* plants are on the right side of each panel. (A) Eight day-old seedlings on MS plates; Note that *sr45-1* has narrow true leaves. (B) Twenty-day-old plants in the soil; several leaves in the *sr45-1* plant are curled downward and inward, indicated by arrow-heads. Inset graph in (B) shows the size of *sr45-1* compared to WT. (C) Thirty-five-day old plants in soil. (D) Fifty-four-day-old plants in soil; note that WT has completed flowering and most of the siliques have turned brown, whereas, the *sr45-1* plant is still flowering. (E) Root growth in *sr45-1* and WT plants. A representative photograph of *sr45-1* and WT seedlings (27 days after germination) illustrates reduced root growth. (F) Quantification of root growth. Each data point is the mean±SEM of 5 plants.

For phenotypic characterization, *sr45-1* and WT plants were grown under identical conditions. At the seedling stage, the leaves of the *sr45-1* plants were elongated and curly (Figure 4A and B). In general *sr45-1* plants were smaller (about one-third) than WT throughout their life cycle (Figure 4A–D). The mutant showed delay in transition to reproductive phase (Figure 4C and D). Root growth was also slower in the mutant as compared to the WT (Figure 4E and F). Under most conditions *sr45-1* plants were bushy (Figure 4D). The most pronounced phenotypes, flowering time and leaf morphology were chosen for further detailed characterization.

### *sr45-1* Plants Flowered Later than WT in Both Long-day and Short-day Photoperiods

**Figure 5:**
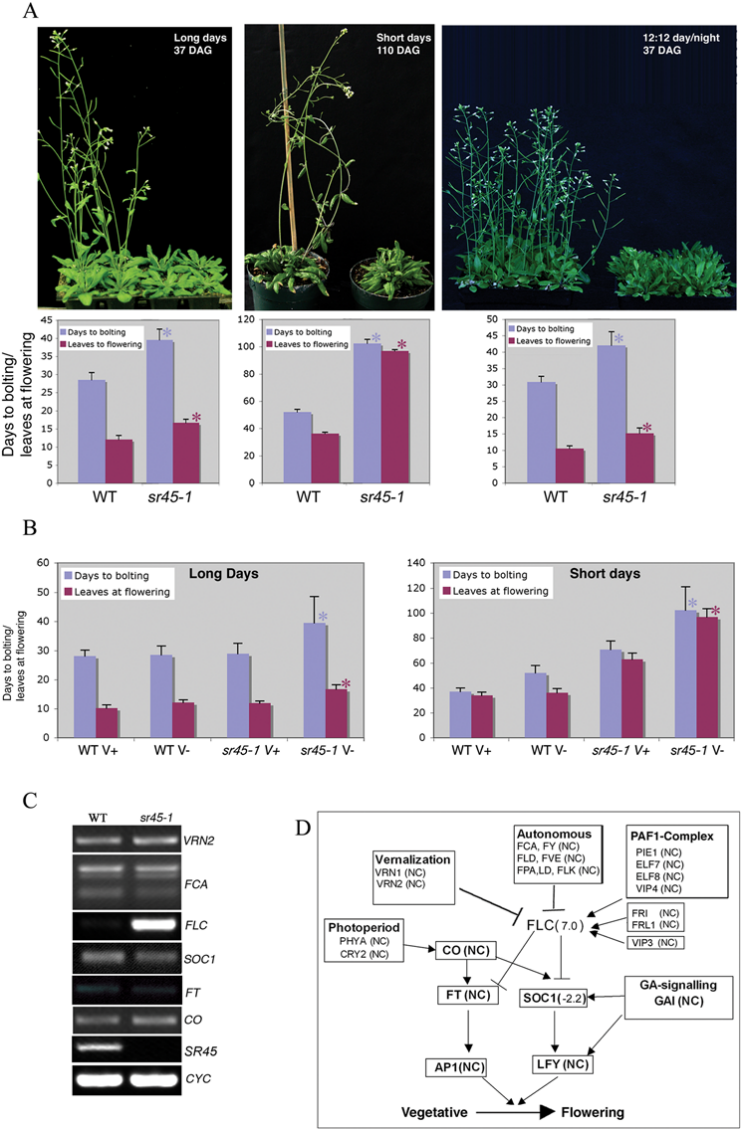
sr45-1 plants are late flowering. (A) Top panel: *sr45-1* plants are considerably later flowering than WT under long-day (LD, 16 h∶8 h light∶dark), short-day (SD, 8 h∶16 h light∶dark) and 12 h∶12 h light∶dark conditions. Age of the plants at the time they were photographed is indicated on each panel. Bottom Panel: Quantification of flowering time. Transition to reproductive stage was measured as days to bolting and number of rosette leaves at the appearance of 1^st^ flower. Each bar is the mean±SEM of 48 to 72 plants. Significant differences (*p*<0.05) between WT and *sr45-1* plants are indicated by ‘*’. Each experiment was repeated three times. (B) Effect of vernalization on flowering of *sr45-1* plants. *sr45-1* and WT seeds were stratified at 4°C for 2 days or vernalized for 40 days at 4°C and grown under LD (16 h photoperiod) or SD (8 h photoperiod) conditions in soil. Flowering time was measured as described in experimental procedures. V+and V-indicate vernalized and unvernalized plants, respectively. Significant differences (*p*<0.05) between vernalized and unvernalized plants are indicated by ‘*’. Each experiment was repeated three times. (C) Expression analyses of flowering related genes in *sr45-1* and WT plants. Expression of representative genes in various flowering pathways was analyzed by RT-PCR. *VRN2* belongs to the vernalization pathway, *FCA* to the autonomous pathway; *FLC* integrates signals from these two pathways. *CO* belongs to the photoperiod pathway. *SOC1* and *FT* function downstream of *FLC*. Each RT-PCR was repeated at least three times. (D) Expression levels of various genes in the flowering time pathways of Arabidopsis. Model is adapted from [24], [70]. Value in parentheses next to a gene indicates induction or repression of that gene in *sr45-1*. NC, No change.

Genetic analysis of flowering time in Arabidopsis has uncovered four major flowering pathways–the photoperiodic, the vernalization, the autonomous and the hormonal pathways [23], [24]. To determine which of these pathways is affected in *sr45-1*, we quantified flowering time under short-day (SD, 8 h:16 h light:dark) and long-day (LD, 16 h∶8 h light∶dark) conditions. Flowering time was measured as days to bolting and number of rosette leaves at the first appearance of a flower. Under LD a majority of WT plants flowered around 29 days after germination (Figure 5A). In contrast, *sr45-1* plants flowered around 40 days, reflecting, on average, 11 days of delay in flowering compared to the WT (Figure 5A). Similarly, at the appearance of the first flower, *sr45-1* had 17 leaves whereas WT had only 12 leaves. Under SD for *sr45-1*, the average number of days to bolting was 102, which was about twice the time required for WT to start bolting (Figure 5A). Likewise, the number of leaves at flowering was 97 in *sr45-1* as compared to 36 in WT. Flowering time under a 12-h photoperiod was very similar to LD (Figure 5A). Together, these data indicate that *sr45-1* exhibits delayed flowering under both LD and SD, although the delay is more prominent under SD.

### The Late Flowering Phenotype of *sr45-1* Was Rescued by Vernalization but Was Insensitive to Photoperiod, Placing it in The Autonomous Pathway of Flowering

To determine the effect of vernalization on the flowering of *sr45-1*, we vernalized *sr45-1* and WT seeds for 40 days and grew them under LD and SD conditions. Under LD, in WT vernalization marginally decreased days to bolting by one day and number of leaves at flowering from 12 to 10. On the other hand, vernalization of *sr45-1* reduced the number of days to bolting and leaves at flowering close to WT (Figure 5B). Similarly under SD, vernalization also reverted flowering time closer to WT. However, unlike under LD, vernalization was not fully effective. Vernalized *sr45-1* plants flowered 31 days earlier than unvernalized plants, however, they were still 34 days later in flowering than vernalized WT plants, indicating a slight interaction of photoperiod with vernalization on flowering. In summary, the SR45-dependent late flowering phenotype was rescued by vernalization, demonstrating that SR45 affects the autonomous pathway.

To gain insight into the molecular effects of SR45 on flowering, we performed microarray experiments consisting of three biological replicates of *sr45-1* and WT using the Affymetrix Arabidopsis 22K gene chip and analyzed the data for flowering time genes (Table S1 and Figure S1). One of the prominent genes that displayed increased expression in *sr45-1* was the *FLOWERING LOCUS C* (*FLC*) (Figure 5C, Figure S1B and Table S1), which is a MADS-box transcription factor and was originally identified as a mutation that was early flowering under LD [25], [26]. The *FLC* gene is a convergence point for several flowering pathways [23], [24]. Close to a dozen genetic loci that affect the expression of *FLC* have been characterized phenotypically and at the molecular level [23], [24]. Seven of these genes, *FCA* [27], *FY* [28], *FVE* [29], *FLD* [30], *LD* [31], *FLK* [32], [33] and *FPA* [34] belong to the autonomous pathway and three (*VRN1, VRN2*, and *VIN3)* belong to the vernalization pathway (Figure 5D). Expression analyses of these genes in the microarray expriment combined with verification by RT-PCR showed that these genes were not differentially expressed in *sr45-1* as compared to WT (Figure 5D and Figure S1 and Table S1). *VRN2* [35] and *FCA*, which is known to regulate flowering by producing multiple transcripts [27], did not reveal any distinguishable alteration in their expression level or AS pattern between *sr45-1* and WT (Figure 5C and Figure S1). These analyses, however, do not rule out the possibility that subtle changes in the AS, limited to only a few nucleotides and indistinguishable by RT-PCR, are affected by SR45. Similarly, the AS pattern of *FLC* gene was also unchanged in *sr45-1*. FLC represses the downstream target genes *SOC1* [36] and *FT* [37]. RT-PCR analyses showed no differential expression of *FT*, whereas there was some repression of *SOC1* in *sr45-1* compared to WT (Figure 5C and D), suggesting that the elevated level of *FLC* in *sr45-1* is causing late-flowering phenotype by affecting the activity of some other downstream components. Microarray and RT-PCR analyses with the *CONSTANS (CO)* gene (photoperiod pathway) [38], showed no alteration of expression in *sr45-1* indicating that it does not affect the photoperiod flowering pathway. Similarly, the expression of several genes that positively regulate *FLC*, such as the members of the PAF1-complex, were also not changed (Figure S1 and Table S1). Overall, these analyses indicate that SR45 affects flowering in an *FLC*-dependent manner.

### Characterization of Leaf Morphology

**Figure 6:**
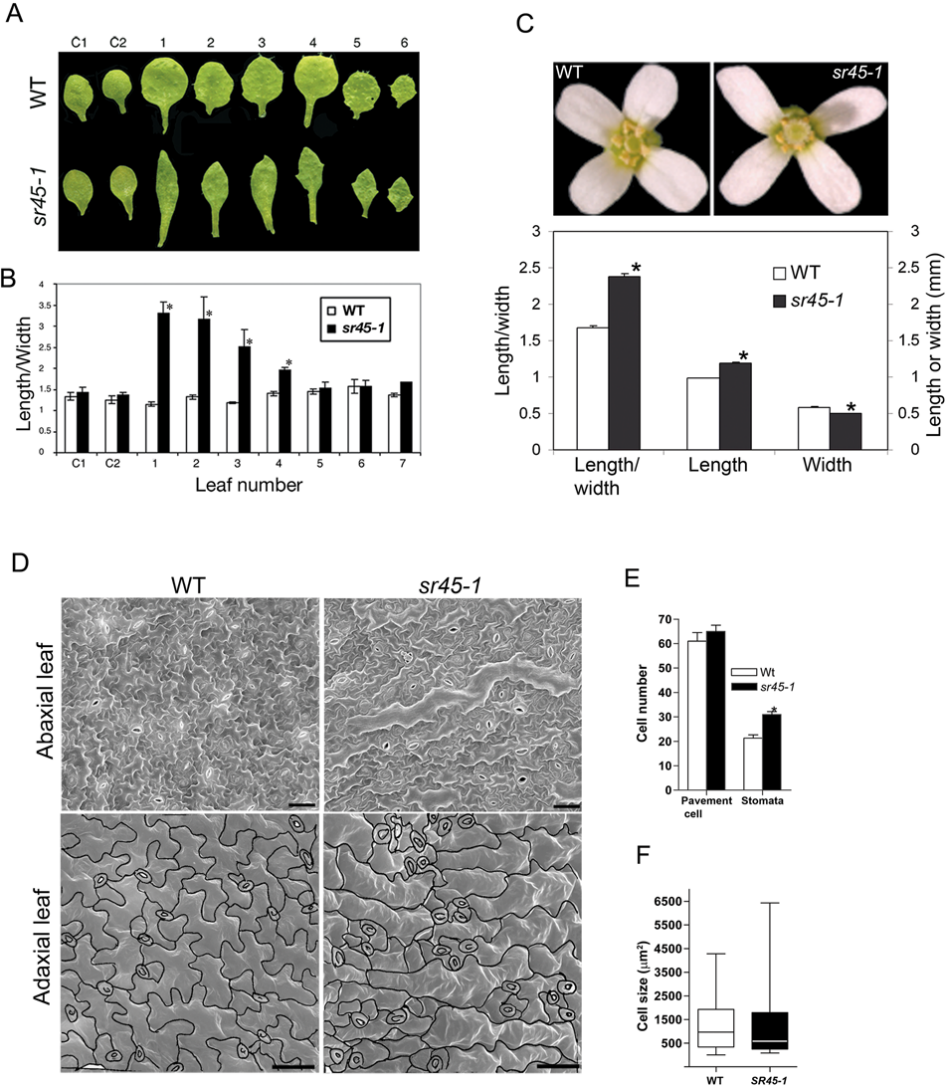
*sr45-1* plants have elongated leaves and petals. (A) WT and *sr45-1* plants grown on MS plates. Note that *sr45-1* has elongated leaves. Cotyledonary leaves (C1 and C2) and true leaves are numbered from left (older) to right (younger). (B) Leaf length/width ratios (Mean±SEM) of WT and *sr45-1* plants. (*n* = 11 leaves from 11 plants, Student's *t*-test *p*-values are as follows: Cotyledon (C1), *p* = 0.52; Cotyledon (C2), *p* = 0.39; leaf 1, *p* = 0.002; leaf 2, *p* = 0.021; leaf 3, *p* = 0.022; leaf 4, *p* = 0.0003; leaf 5, *p* = 0.33; leaf 6, *p* = 0.49; leaf 7, *p* = 0.29). The symbol ‘*’ on the top of bars indicate that the difference between WT and *sr45-1* is significant (*p*<0.05). (C) Fully opened flowers of *sr45-1* plants have elongated petals. The bottom panel shows quantification of length, width and length/width ratio (Mean±SEM) of petals in WT (WT) and mutant (*sr45-1*) plants (*n* = 16, Student's *t*-test *p*-values: length, *p* = 10^−9^, width, *p* = 10^−8^ *;*length/width *p* = 10^−10^). The symbol ‘*’ on the top of bars indicate that the difference between WT and *sr45-1* is significant (*p*<0.05). (D) The leaves of *sr45-1* plants exhibit abnormal expansion of pavement cells. Scanning electron micrographs of the abaxial and adaxial surfaces of WT and *sr45-1* plants. Note the presence of abnormal elongated abaxial pavement cells in *sr45-1*. For clarity, the boundaries of pavement cells on the adaxial surfaces are outlined. Note the presence of markedly enlarged and elongated pavement cells in *sr45-1* leaves. Bars = 50 µm. (E) Quantitative analyses of the number of pavement cells and stomata in WT and *sr45-1*. Each bar is the average±SEM of cells counted in at least three 160 mm^2^ areas in three different leaves. The *p*-values of the *t*-test statistics of pavement cell and stomata were 0.55 (non-significant) and 0.03 (significant, indicated by ‘*’), respectively. (F) Box plots of the distribution of cell sizes in WT and *sr45-1* pavement cells. Each box plot was made from the cell sizes of WT (*n* = 112) and *sr45-1* (*n* = 128) from at least three different leaves. Boxes indicate the interquartile range between the 1st and 3rd quartile, whereas line in the middle of the box represents median. Ends of the vertical lines indicate the range of the data.

To quantify the change in leaf shape, we analyzed the length to width ratio of leaves in WT and *sr45-1*. Significant differences in the leaf length/width ratio were observed between WT and *sr45-1*, which were more obvious in juvenile (leaf 1 to 4) than in mature leaves (leaf 5 to 7) (Figure 6A and B). On average *sr45-1* leaves were about two-fold more elongated than in WT. Since floral organs are modified leaves, we also compared their morphology in fully opened flowers of *sr45-1* and WT. Like vegetative leaves, petals were also elongated in *sr45-1* (Figure 6C). Together, these data indicate that SR45 is necessary for the normal expansion of organs.

To better characterize the leaf morphology, we compared epidermal cell-shape in the leaves of *sr45-1* to WT with scanning electron microscopy (SEM). Compared to WT, the abaxial surfaces of *sr45-1* leaves had some pavement cells that were abnormal in shape (Figure 6D). These cells were significantly more elongated along the length of the leaf and did not display clear indentations and lobes as in WT cells. The presence of these cells probably interferes with normal expansion of leaf cells resulting in the curly phenotype of *sr45-1* leaves. Comparison of the adaxial surfaces of *sr45-1* to WT revealed two distinct phenotypes. First, several pavement cells of *sr45-1* displayed abnormal expansion along all axes. In these cells, the indentations and lobes, although detectable, were less prominent as compared to WT making them look more elongated and arranged length-wise along the length of leaf (Figure 6D). Second, contrary to regularly spaced stomata in WT leaves, in *sr45-1* they were clustered together. To quantify the contribution of cell size and cell number to the elongated phenotype of leaves, we compared the number of pavement cells and stomata in *sr45-1* to WT. Stomata were approximately 30% more in *sr45-1* than in WT (Figure 6E). The number of pavement cells per unit area in *sr45-1* was slightly more than in WT (65/mm^2^ in *sr45-1* to 61/mm^2^ in WT), however this difference was statistically not significant (*p* = 0.55) (Figure 6E). The fact that there are more stomata in the mutant suggests that cell division may have been affected early in leaf development. Cell size and the orientation of expansion on the contrary were dramatically changed in *sr45-1*. Although, the means of pavement cells size of *sr45-1* and WT were not significantly different (*p* = 0.3), visual examination revealed obvious differences in the distribution of their cell sizes. Quantification of the cell size data is displayed as box plots in Figure 6F, which show that the cell size distribution in *sr45-1* is more varied than in WT (size range was 6347 mm^2 ^in *sr45-1* compared to 4199 mm^2^ in WT). Additionally, the scatter of cell sizes in the 4^th^ quartile was about two-fold greater for *sr45-1* than for WT. This shows that a substantial proportion of *sr45-1* cells were bigger than the biggest cells in WT. Together, these data indicate that the elongated leaf shape phenotype is probably caused by abnormal cell expansion and by subtle changes in cell division.

### Flowers of the *sr45-1* Plants Have Abnormal Petal and Stamen Numbers

**Figure 7:**
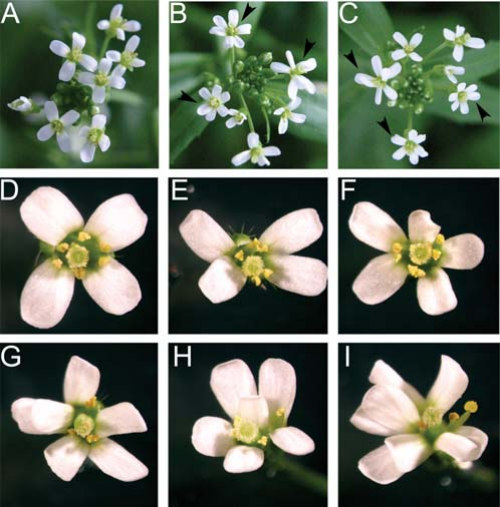
*sr45-1* Flowers have Altered Number of Sepals and Petals. (A) WT inflorescence shows that all flowers had four petals. (B and C) Inflorescences from two separate *sr45-1* plants are shown. Each panel shows 3 of 6 flowers with abnormal petal and stamen numbers, indicated by arrowhead. (D) Close-up of a WT flower with 4 petals 6 stamens. (E–I) *sr45-1* flowers with abnormal petal and stamen numbers. Number of sepals and carpels were not changed. (E) 4 petals 5 stamens (F) 5 petals 5 stamens (G) 6 petals 3 stamens (H) 6 petals 4 stamens (I) 8 petals 3 stamens.

The mutant plants grown under short day conditions had higher occurrence of flowers with altered number of petals and stamens (Figure 7). This phenotype was more prominent in the early flowers and was rarely observed in later flowers. This phenotype is different from the well-studied phenotype of mutants of floral homeotic genes, where whole whorls of floral organs change from one type to another. Instead, *sr45-1* flowers have all four whorls but the number of petals and stamens varied from 3 to 8 in several combinations (Figure 7B–I). The number of sepals and carpels remained unchanged. In the same inflorescence several flowers had the WT organ numbers (Figure 7B and C). However, most of the inflorescences had at least one flower with altered petal and stamen numbers. Overall, of the 67 flowers counted, 11 had the abnormal petal and stamen number, which corresponds to a penetrance of approximately 15%. SR45 thus partially affects the petal and stamen number in a development-specific manner.

### Full-length *SR45* Rescued *sr45-1*

**Figure 8:**
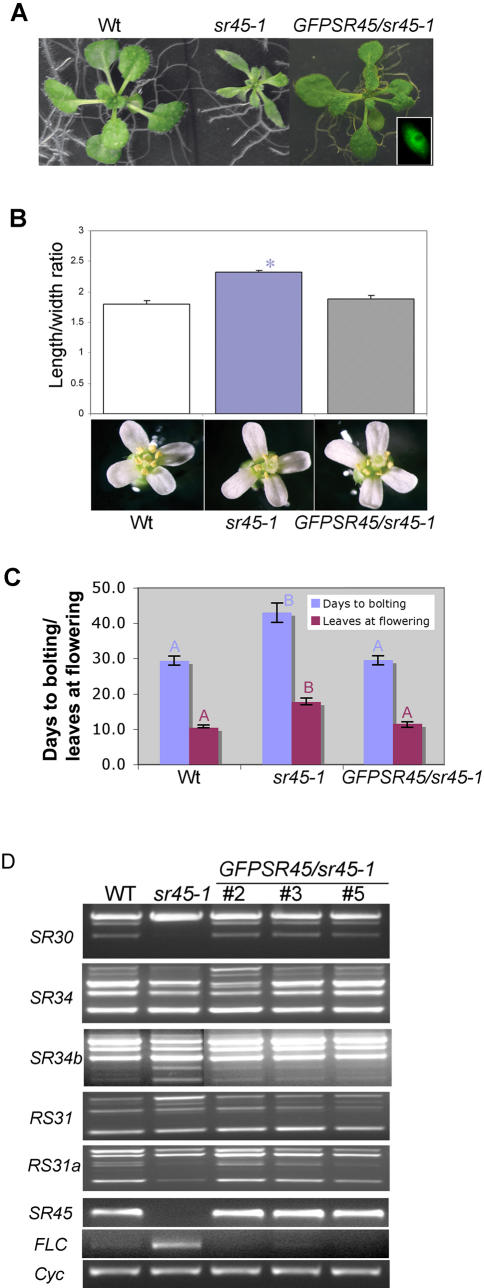
Full-length *GFP*-tagged *SR45* rescued the mutant phenotypes of *sr45-1* plants. (A) Growth of WT, *sr45-1* and *GFP-SR45*/*sr45-1* plants. The narrow-leaf and stunted growth phenotype of *sr45-1* was rescued by expression of *GFP-SR45* in *sr45-1* plants. Inset in the lower right corner shows a root epidermal nucleus of a *GFP-SR45*/*sr45-1* displaying the characteristic speckled distribution of GFP-SR45. (B) Petal shape phenotype of WT, *sr45-1* and *GFP-SR45*/*sr45-1*. The *p*-values of *t*-test statistics are as follows: WT vs *sr45-1*, *p* = 0.000012 (significant); WT vs *GFPSR45/sr45-1*, *p* = 0.32 (non-significant). Significant difference is indicated by ‘*’. (C) Quantification of flowering time. Transition to reproductive stage was measured as days to bolting and number of rosette leaves at the appearance of 1^st^ flower. Each bar is the mean±SEM of 10 to 22 plants. Bars with same letter and the same color indicate non-significant difference (*p*>0.05). (D) Expression pattern of alternatively spliced transcripts of *SR* genes in WT, *sr45-1* and *GFP-SR45*/*sr45-1* plants. Expression pattern of three independent *GFP-SR45*/*sr45-1* lines is shown. In all transgenic lines the splicing patterns of pre-mRNAs encoding SR proteins is restored to wild-type. Similar results were obtained in two independent RT-PCR analyses. For details on RT-PCR and primer sets used see Figure 3. Cyclophilin amplification was used to demonstrate equal amount of template in each PCR.

To demonstrate that the lack of SR45 was responsible for the *sr45-1* phenotype, we complemented the mutant with a *GFP*-tagged *SR45* (*GFP-SR45*). The *GFP-SR45* construct was described earlier and was shown to be functional [20]. These plants exhibited a characteristic speckled pattern of GFP-SR45 in the nuclei indicating that it was expressed and localized correctly (Figure 8A, inset). The ectopic expression of *GFP-SR45* in transgenic *sr45-1* plants reverted their narrow-leaf, narrow-petal and flowering phenotype back to the WT (Figure 8A, B and C). Consistent with the flowering time of the rescued *GFP-SR45/sr45-1* plants, the *FLC* levels were also reverted back to WT (Figure 8D, *FLC*), further confirming that the mutant phenotypes were due to *SR45* mutation. Similarly, RT-PCR analysis of several independent transformants showed the expected size *SR45* product (Figure 8D, *SR45*). In addition, the splicing pattern of *SR30, SR34, SR34b, RS31* and *RS31a* was also rescued in three independent *GFP-SR45/sr45-1* transgenic lines (Figure 8D).

## Discussion

### SR45 Is a Plant-Specific Splicing Factor

SR45 was identified as a U1-70k interacting protein in a yeast two-hybrid screen [17] and verified to co-localize with U1-70K and another splicing factor, SR1, in the nucleus as speckles [20], a hallmark feature of all SR proteins both in plants and metazoans [10], [39]. These observations, together with its unique domain organization (an N-terminal and a C-terminal RS domain separated by an RRM domain), suggested that SR45 plays a role in splicing (Figure 2). To be classified as a bonafide splicing factor, a protein is usually tested for its ability to complement splicing in a splicing-deficient extract. We showed that SR45 was able to splice a pre-mRNA substrate in a splicing-deficient extract, suggesting that it is a splicing factor (Figure 1B). This finding is further supported by the fact that a mutation in *SR45* led to changes in the splicing pattern of several other SR genes (discussed below).

### SR45 Is Involved in Plant-Specific Developmental Processes

Homologs of SR45 are found only in higher plants, which suggests, that SR45 performs a plant-specific function. This notion is supported by the functional characterization of SR45 at the phenotypic and molecular levels. The most prominent plant-specific function affected by SR45 was flowering. The *sr45-1* plants were late flowering in both SD and LD conditions, essentially placing it in the autonomous pathway. This was validated by physiological, molecular and genetic analyses. First, the late-flowering phenotype was overcome by vernalization under LD (Figure 5) consistent with the late-flowering characteristics of other autonomous pathway mutants such as *fve, fld, fca, fy, fpa, ld* and *flk,* all of which are late flowering under both SD and LD and are rescued by vernalization [28]–[34]. Second, *sr45-1* had an elevated level of *FLC* (Figure 5C, Figure S1B), whose expression is elevated in all autonomous pathway mutants. Third, expression of *CO*, which is a photoperiod pathway gene, was not changed in the *sr45-1* mutant, indicating that SR45 does not operate in the photoperiod pathway.

RT-PCR analyses showed that a truncated transcript of *SR45* is produced in *sr45-1* plants, which may code for a truncated version of SR45 that would lack the last one-third of the protein. This may result in a non-functional or a protein with abnormal function ultimately resulting in impaired activity in splicing. However, the level of the truncated transcript in the mutant is very low (about 8% of WT level, Fig. 2D). In support of this conclusion, the heterozygous plants were similar to WT (data not shown). Hence, the observed phenotypes in *sr45-1* plants are not likely due to a hypomorphic allele or a dominant negative allele of SR45.

Arabidopsis leaf development requires the establishment of proximodistal, adaxio-abaxio and mediolateral asymmetry, which is a complex process controlled by several genes that are involved in polar cell expansion and differentiation [40], [41]. The leaf morphology of *sr45-1* bears resemblance to those mutants that are defective in the leaf length-width expansion. Analysis of our microarray and RT-PCR data for several known genes involved in leaf morphology such as *ANGUSTIFOLIA* (*AN*), *ROT3* [40], *AN3* [42], *CURLY LEAF* (*CLF*) [43], *DRL* [44], *AS1* [45], *AS2* [46] and *ATHB13* [47], however, did not reveal a significant change in their expression level between the mutant and WT (Figure S2 and Table S2). This indicates that these genes are not affected by SR45. Comparison of cell shapes and number in *sr45-1* to WT showed that SR45 likely affects both cell expansion and division. Several genes involved in diverse cellular processes affect cell expansion. For example, wall-associated kinase2 (*WAK2*) antisense plants had smaller cell sizes than control plants [48]. Interestingly, in *sr45-1* leaves, which had more expanded cells, the expression of this gene (259560_at) together with several other WAK-like genes such as, *WAK1* (259561_at) and *WAK3* (259559_at) were moderately (2 to 3-fold over WT) induced (Table S2, and RT-PCR data not shown) suggesting a positive correlation between the expression of these genes with cell expansion. The stomatal density phenotype in *sr45-1* showed resemblance to the stomatal density mutants such as *tmm* [49] *sdd* [50] *yda* [51] and a synergistically interacting group of receptor-like kinases, *er erl1 erl2* [52]. However, an analysis of the expression of these genes in the microarray data did not reveal significant differences between *sr45-1* and WT plants, indicating that SR45 acts on yet to be identified genes for controlling stomatal density. Our work revealed the importance of splicing in development and provides a framework for investigating the role of splicing in the development of leaves and other organs.

In addition to late-flowering phenotypes, *sr45-1* plants also had defects in the number of petals and stamens in a significant proportion of plants (Figure 7). The abnormal organ number observed in *sr45-1* did not follow the well-studied characteristic ABC model, which specify organ identity in each of the four whorls of a flower rather than their number in each whorl [53]. Therefore, SR45 likely controls the activities of gene(s) involved in specifying the number of floral organs. Altered organ numbers in the Arabidopsis flowers is observed in lesions in several genes, such as *WIGGUM/ERA1* [54], *ULTRAPETALA* [55], *PLURIPETALA* [56], *PIE1* [57], *CLV1* [58], *CLV2* [59] and *CLV3* [60]. None of these genes were, however, differentially expressed (Figure S2 and Table S2), suggesting that other genes, which may or may not be related to the reported genes, may be affecting the organ numbers in an SR45-dpenedent manner.

### RNA Targets of SR45 Include Other SR Genes

SR45 is a splicing factor and therefore would affect a phenotypic outcome by affecting splicing of other genes. An effect on constitutive splicing could lead to the accumulation of unspliced pre-mRNAs, which may encode truncated proteins and/or have reduced stability. Transcripts of plant SR genes themselves are the targets of AS regulation in a tissue-and development-specific manner [61]–[64]. To understand the mechanism of SR45 in splicing, we analyzed the AS pattern of all SR genes in *sr45-1* and WT plants. Analyses of SR genes showed changes in the AS pattern of several SR genes, which in turn are likely to regulate the splicing of other genes. In affected SR genes, the longest intron was alternatively spliced. In most cases, the general pattern is a reduction in the usage of the distal 3′ splice sites that generate a smaller transcript and an increase in the usage of the proximal 3′ splice sites in *sr45-1* compared to WT. This indicates that the WT SR45 protein favors the usage of the distal 3′ AS sites and is responsible for a specific balance of alternative transcripts. Although the effect of depleting a specific plant SR protein on the splicing pattern of other genes has not been reported so far, studies with overexpression of two different types of plant SR proteins, SR30 and RSZ33, resulted in abnormalities in development and morphology, which were accompanied by a marked change in the splicing pattern of *SR30* and *SR1* [62], [64]. These studies indicate that the ratio of the splice variants is critical for normal plant development. SR45 also changed the splicing pattern of transcripts from several other SR genes. This, together with the fact that SR45 complements S100 cell extract, suggests that it is involved in both constitutive and regulated splicing. Based on our observation that the AS pattern of several other SR genes is differentially modulated by SR45, it is likely that the splice variants of SR proteins modulate the splicing and/or other RNA processing activities of genes involved in the developmental processes affected in the *sr45-1* plants.

## Materials and Methods

### Plant Material and Growth Conditions

Seeds of T-DNA insertion lines in the *SR45* (At1g16610) in Columbia (Col) background were obtained from the SALK collection (http://signal.salk.edu/; Salk_004132) at the ABRC. The T-DNA insert was verified by genomic PCR with *SR45*-specific and the T-DNA-specific primers. WT and homozygous mutant plants were grown under identical conditions of 100 µmoles m^−2^ s^−1^, 16 h∶8 h dark∶light cycle at 22°C and 70% RH. Seeds from fully mature siliques were collected and used in subsequent experiments. Seeds from WT and *sr45-1* were surface sterilized, suspended in 0.1% Phytagar and stratified for 2 days at 4°C and depending on the experiment plated on Murashige and Skooge (MS) plates (MS salts, 1% sucrose, 0.8% Phytagar) or directly sown in soil. Root length of WT and *sr45-1* plants was measured on every second day. Size of twenty-day-old WT and *sr45-1* plants was determined from photographs with the NIH Image J (http://rsb.info.nih.gov/ij/). In determining the relative size of *sr45-1*, WT size was considered as 100 percent.

### Database searches and amino acid sequence alignment

Arabidopsis (www.arabidopsis.org), rice (*Oryza sativa* cv. *japonica*, http://cdna01.dna.affrc.go.jp/cDNA/), algae (*Chlamydomonas reinhardtii,* http://genome.jgi-psf.org/Chlre3/Chlre3.home.html; *Cyanidioschyzon merolae* , http://merolae.biol.s.u-tokyo.ac.jp/) a diatom (*Thalassiosira pseudonana,* http://genome.jgi-psf.org/thaps1/thaps1.home.html), fission yeast (*Schizosaccharomyces pombe,* http://www.sanger.ac.uk/Projects/S_pombe/), budding yeast (*Saccharomyces cerevisiae*, http://www.yeastgenome.org/), human (*Homo sapiens*, http://www.ensembl.org/), *Caenorhabditis elegans* (www.wormebase.org), fruit fly (*Drosophila melanogastor*, www.flybase.org) and Eukaryotic Gene Orthologs [EGO; http://compbio.dfci.harvard.edu/tgi/cgi-bin/tgi/gimain.pl?gudb = maize]) databases were searched with the Arabidopsis SR45 protein sequence using BLASTP and TBLASTX. Downloaded sequences were analyzed for the presence of RRM using Interproscan (http://www.ebi.ac.uk/InterProScan/) and the presence of RS domain manually. The amino acid sequences of Arabidopsis (At1g16610), rice (AK070420 and AK063761) and maize (translated from BT016650) SR45 proteins were aligned with ClustalX using default parameters [65].

### In Vitro Splicing Assay

The coding region of *SR45* was amplified from a cDNA clone with forward (5′CG**GGATCC**GCGAAACCAAGTCGTGGC3′) and reverse primers (5′CCG**CTCGAG**TTAAGTTTTACGAGGTGGAG3′) containing BamHI and XhoI sites (indicated in bold), respectively. The amplified product was digested with BamHI and XhoI and ligated into pFASTBAC vector and verified by sequencing. The recombinant baculovirus was prepared as recommended by the manufacturer (Invitrogen). Recombinant virus was used to infect Sf21 cells and the recombinant protein fused to His.tag was purified using Ni^2+^ affinity chromatography [66]. ASF/SF2, which was also fused to His.tag and expressed in insect cells, was purified essentially as SR45. For in vitro S100 complementation splicing assays, 10, 30 and 90 ng of SR45 was used with the β-globin pre-mRNA as a substrate. Twenty-five ng of His-tagged ASF/SF2 was used as a positive control. Splicing assays were carried out for 2 hours at 30°C. Splicing reactions were deproteinized and precipitated with ethanol. Spliced products were fractionated in a 6% denaturing PAGE and visualized by autoradiography.

### Quantification of Leaf and Petal Morphology

Cotyledonary and rosette leaves from 3 week old WT and *sr45-1* plants grown on MS plates were excised, arranged in the order of age and photographed together. Length and width of each leaf was determined using Adobe Photoshop and the length/width ratio was calculated. Similarly, fully-opened flowers from soil-grown WT and *sr45-1* plants were photographed and the length/width ratio of petals was determined. Data were statistically analyzed for significant differences using the Students' *t*-test in Microsoft Excel.

### Flowering Time Measurement and Vernalization Treatment

For vernalization, seeds were surface-sterilized, suspended in 0.1% Phytagar and stratified for 2 days at 4°C. Seeds were incubated at 22°C for 2 days and transferred back to 4°C for another 40 days. Unvernalized seeds were surface-sterilized and stratified for 2 days at 4°C. Vernalized and unvernalized seeds were germinated and grown under LD (16 h∶8 h light∶dark) or SD (8 h∶16 h light∶dark) conditions. Flowering time was measured both as days to bolting and the number of rosette leaves at flowering. Data for 48 to 72 plants were statistically analyzed for significant differences using the Students' *t*-test in Microsoft Excel.

### RT-PCR Analyses of Flowering Genes and Analysis of Alternative Splicing of Pre-mRNAs of SR Proteins

For flowering time, leaf morphology and floral organ number, RT-PCR analyses were performed with cDNA prepared from 2-week old Arabidopsis seedlings with Takara EX Taq™ polymerase and gene specific primers (Sequences of the primers are provided in Table S3). PCR products were quantified with the NIH Image J software. The intensities of PCR products were normalized to cyclophilin. For determining the AS patterns of the 19 Arabidopsis SR genes, RT-PCRs with gene-specific primers were performed with total RNA isolated from root, stem, leaf and inflorescence of five-week old WT and *sr45-1* plants. Equal amount of template in each assay was verified with cyclophilin primers. The sequences of SR gene primers, which in most cases corresponded to the first and last exons, are given in Table S3.

### Microarray Experiments and Data Analyses

Total RNA was isolated from fifteen-day old seedlings grown on MS plates at 16h∶8h light∶dark cycle and 22°C according to the Trizol method (Invitrogen). The RNA samples were treated with DNAseI and purified using the Qiagen RNA isolation columns. Complementary RNA synthesis, hybridization to the Affymetrix Arabidopsis Genome ATH1 Array, data acquisition, processing and analyses were done according to the Affymetrix GeneChip Expression Analysis Technical Manual (Affymetrix, Santa Clara, CA). Two additional experiments using similar conditions and GeneChip analyses as above were repeated several months apart as a biological replicate. These three experiments generated three control and three *sr45-1* data sets. Each data set was initially analyzed separately with the Affymetrix MAS5.0 software, which yielded log ratios of *sr45-1* signal to WT and an associated *p*-value indicating the significance of the change. For statistical comparison analyses, gene expression data were calculated using the model-based expression index-perfect match (MBEI-PM) algorithm with invariant set normalization in the dChip software (http://www.dchip.org) [67]. The Gene expression data were analyzed for significant differential expression with a moderated paired *t*-test in the R/Bioconductor software (http://www.R-project.org). The resultant *p*-values were corrected for false discovery rate (FDR) using the Benjamini-Hochberg (HB) algorithm in R/Bioconductor [68]. Genes with at least two-fold change with an associated FDR-corrected *p*-value≤0.05 were considered significantly changed in *sr45-1* relative to WT.

### Scanning electron microscopy

Scanning electron microscopy was conducted as described [69]. Cell sizes were measured with the NIH Image J software in at least three SEM images of WT and *sr45-1* leaves.

## Supporting Information

Figure S1 Expression analyses of flowering time genes in WT and *sr45-1* plants. (A) RT-PCR was performed with total RNA isolated from two week old plants with gene-specific primers as described in the Experimental procedures. For full name of genes see the legend of Figure S1B below. (B) PCR bands were quantified with NIH Image J software (http://rsb.info.nih.gov/ij/). Each PCR product in WT and *sr45-1* was normalized to cyclophilin transcript level in WT and *sr45-1*, respectively. Data shown are the percent of WT, with WT levels adjusted to 100 percent. VRN, The vernalization pathway; Autonomous, The autonomous flowering pathway; PAF1, RNA polymerase II (Pol II) Associated Factor 1-complex; VIP, vernalization independence; Integrator; Floral pathway integrators; Photoperiod, Photoperiod pathway genes; MIG, meristem identity genes. *VRN1,VERNALIZATION 1; VRN2, VERNALIZATION 2; FCA, FCA protein; FY, FY protein; FLD, Flowering Locus D; FVE, FVE protein; LD, LUMINIDEPENDENS; FLK, FLOWERING LATE KH DOMAIN; PIE1, PHOTOPERIOD INDEPENDENT EARLY FLOWERING1; ELF7, EARLY FLOWERING 7; ELF8, EARLY FLOWERING 8; VIP4, VERNALIZATION INDEPENDENCE 4; VIP3, VERNALIZATION INDEPENDENCE 3; FRI, FRIGIDA; FRL1, FRIGIDA-LIKE 1; GAI, GA INSENSITIVE; FLC, FLOWERING LOCUS C; SOC1, SUPPRESSER OF OVEREXPRESSER OF CONSTANS 1; FT, FLOWERING TIME T; CRY2, CRYPTOCHROME2; PHYA, PHYTOCHROME A; AP1, APETELLA 1; LFY, LEAFY; CYC, CYCLOPHILLIN*.
(1.19 MB TIF)

Figure S2 Expression analyses of leaf shape and floral organ number genes in WT and *sr45-1* plants. (A) RT-PCR was performed with total RNA isolated from two week old plants with gene-specific primers as described in the Experimental procedures. Left panel consists of RT-PCR of leaf morphology genes; right panel consists of genes affecting petal numbers. *AS1, ASYMMETRIC LEAVES1; AS2, ASYMMETRIC LEAVES2; AN, ANGUSTIFOLIA; AN3, ANGUSTIFOLIA3; ROT3, ROTUNDIFOLIA3; DRL, DEFORMED ROOTS AND LEAVES 1; ATHB13, HOMEODOMAIN LEUCINE-ZIPPER PROTEIN ATHB13; WIG, WIGGUM; ULT, ULTRAPETALLA; CYC, CYCLOPHILLIN*. (B) PCR bands were quantified with NIH Image J software (http://rsb.info.nih.gov/ij/). Each PCR product in WT and *sr45-1* was normalized to cyclophilin transcript level in WT and *sr45-1*, respectively. Data shown are the percent of WT, with WT levels adjusted to 100 percent.
(0.74 MB TIF)

Table S1 Summary of Microarray Analyses of Flowering Time Genes in *sr45-1* and WT Arabidopsis. Gene expression data of *sr45-1* and WT consisting of three biological replicates were statistically analyzed as described in Materials and Methods.
(0.11 MB DOC)

Table S2 Gene expression analyses of all genes present on the Affymetrix Arabidopsis gene chip.
(3.25 MB XLS)

Table S3 Sequences of gene-specific primers of Arabidopsis SR, flowering pathway and leaf morphology genes.
(0.08 MB DOC)

## References

[1] Reddy AS (2007). Alternative Splicing of Pre-Messenger RNAs in Plants in the Genomic Era.. Annu Rev Plant Biol.

[2] Huang Y, Steitz JA (2005). SRprises along a messenger's journey.. Mol Cell.

[3] Graveley BR (2000). Sorting out the complexity of SR protein functions.. RNA.

[4] Sanford JR, Longman D, Caceres JF, Jeanteur P (2003). Multiple roles of the SR protein family in splicing regulation.. Regulation of alternative splicing.

[5] Reddy ASN (2001). Nuclear pre-mRNA splicing in plants.. CRC Crit Rev Plant Sci.

[6] Shen H, Kan JL, Green MR (2004). Arginine-serine-rich domains bound at splicing enhancers contact the branchpoint to promote prespliceosome assembly.. Mol Cell.

[7] Manley JL, Tacke R (1996). SR proteins and splicing control.. Genes Dev.

[8] Wu JY, Maniatis T (1993). Specific interactions between proteins implicated in splice site selection and regulated alternative splicing.. Cell.

[9] Golovkin M, Reddy ASN (1996). Structure and expression of a plant U1 snRNP 70K gene: alternative splicing of U1 snRNP 70K pre-mRNAs produces two different transcripts.. Plant Cell.

[10] Reddy AS (2004). Plant serine/arginine-rich proteins and their role in pre-mRNA splicing.. Trends Plant Sci.

[11] Kalyna M, Barta A (2004). A plethora of plant serine/arginine-rich proteins: redundancy or evolution of novel gene functions?. Biochem Soc Trans.

[12] Isshiki M, Tsumoto A, Shimamoto K (2006). The serine/arginine-rich protein family in rice plays important roles in constitutive and alternative splicing of pre-mRNA.. Plant Cell.

[13] Iida K, Go M (2006). Survey of Conserved Alternative Splicing Events of mRNAs Encoding SR Proteins in Land Plants.. Mol Biol Evol.

[14] Romac JM, Keene JD (1995). Overexpression of the arginine-rich carboxy-terminal region of U1 snRNP 70K inhibits both splicing and nucleocytoplasmic transport of mRNA.. Genes Dev.

[15] Kohtz JD, Jamison SF, Will CL, Zuo P, Lührmann R (1994). Protein-protein interactions and 5′-splice-site recognition in mammalian mRNA precursors.. Nature.

[16] Golovkin M, Reddy ASN (1998). The plant U1 small nuclear ribonucleoprotein particle 70K protein interacts with two novel serine/arginine-rich proteins.. Plant Cell.

[17] Golovkin M, Reddy ASN (1999). An SC35-like protein and a novel serine/arginine-rich protein interact with Arabidopsis U1-70K protein.. J Biol Chem.

[18] Lopato S, Forstner C, Kalyna M, Hilscher J, Langhammer U (2002). Network of interactions of a novel plant-specific Arg/Ser-rich protein, atRSZ33, with atSC35-like splicing factors.. J Biol Chem.

[19] Ali GS, Reddy AS (2006). ATP, phosphorylation and transcription regulate the mobility of plant splicing factors.. J Cell Sci.

[20] Ali GS, Golovkin M, Reddy AS (2003). Nuclear localization and in vivo dynamics of a plant-specific serine/arginine-rich protein.. Plant J.

[21] Wang BB, Brendel V (2004). The ASRG database: identification and survey of Arabidopsis thaliana genes involved in pre-mRNA splicing.. Genome Biol.

[22] Tacke R, Tohyama M, Ogawa S, Manley JL (1998). Human Tra2 proteins are sequence-specific activators of pre-mRNA splicing.. Cell.

[23] Sung S, Amasino RM (2005). REMEMBERING WINTER: Toward a Molecular Understanding of Vernalization.. Annu Rev Plant Biol.

[24] Boss PK, Bastow RM, Mylne JS, Dean C (2004). Multiple Pathways in the Decision to Flower: Enabling, Promoting, and Resetting..

[25] Sheldon CC, Burn JE, Perez PP, Metzger J, Edwards JA (1999). The *FLF* MADS box gene: a repressor of flowering in Arabidopsis regulated by vernalization and methylation.. Plant Cell.

[26] Michaels SD, Amasino RM (1999). FLOWERING LOCUS C encodes a novel MADS domain protein that acts as a repressor of flowering.. Plant Cell.

[27] Macknight R, Duroux M, Laurie R, Dijkwel P, Simpson G (2002). Functional significance of the alternative transcript processing of the Arabidopsis floral promoter *FCA*.. Plant Cell.

[28] Simpson GG, Dijkwel PP, Quesada V, Henderson I, Dean C (2003). FY is an RNA 3′ end-processing factor that interacts with FCA to control the Arabidopsis floral transition.. Cell.

[29] Ausin I, Alonso-Blanco C, Jarillo JA, Ruiz-Garcia L, Martinez-Zapater JM (2004). Regulation of flowering time by FVE, a retinoblastoma-associated protein.. Nat Genet.

[30] He Y, Michaels SD, Amasino RM (2003). Regulation of flowering time by histone acetylation in Arabidopsis.. Science.

[31] Lee I, Aukerman MJ, Gore SL, Lohman KN, Michaels SD (1994). Isolation of *LUMINIDEPENDENS*: a gene involved in the control of flowering time in Arabidopsis.. Plant Cell.

[32] Mockler TC, Yu X, Shalitin D, Parikh D, Michael TP (2004). Regulation of flowering time in Arabidopsis by K homology domain proteins.. Proc Natl Acad Sci U S A.

[33] Lim MH, Kim J, Kim YS, Chung KS, Seo YH (2004). A new Arabidopsis gene, *FLK*, encodes an RNA binding protein with K homology motifs and regulates flowering time via *FLOWERING LOCUS C*.. Plant Cell.

[34] Schomburg FM, Patton DA, Meinke DW, Amasino RM (2001). *FPA*, a gene involved in floral induction in Arabidopsis, encodes a protein containing RNA-recognition motifs.. Plant Cell.

[35] Gendall AR, Levy YY, Wilson A, Dean C (2001). The VERNALIZATION 2 gene mediates the epigenetic regulation of vernalization in Arabidopsis.. Cell.

[36] Lee H, Suh SS, Park E, Cho E, Ahn JH (2000). The AGAMOUS-LIKE 20 MADS domain protein integrates floral inductive pathways in Arabidopsis.. Genes Dev.

[37] Kardailsky I, Shukla VK, Ahn JH, Dagenais N, Christensen SK (1999). Activation tagging of the floral inducer FT.. Science.

[38] Putterill J, Robson F, Lee K, Simon R, Coupland G (1995). The *CONSTANS* gene of Arabidopsis promotes flowering and encodes a protein showing similarities to zinc finger transcription factors.. Cell.

[39] Lamond AI, Spector DL (2003). Nuclear speckles: a model for nuclear organelles.. Nat Rev Mol Cell Biol.

[40] Tsukaya H (2006). Mechanism of Leaf Shape Determination.. Annu Rev Plant Biol.

[41] Byrne ME (2005). Networks in leaf development.. Curr Opin Plant Biol.

[42] Horiguchi G, Kim GT, Tsukaya H (2005). The transcription factor AtGRF5 and the transcription coactivator AN3 regulate cell proliferation in leaf primordia of Arabidopsis thaliana.. Plant J.

[43] Kim GT, Tsukaya H, Uchimiya H (1998). *The CURLY LEAF* gene controls both division and elongation of cells during the expansion of the leaf blade in *Arabidopsis thaliana*.. Planta.

[44] Nelissen H, Clarke JH, De Block M, De Block S, Vanderhaeghen R (2003). DRL1, a homolog of the yeast TOT4/KTI12 protein, has a function in meristem activity and organ growth in plants.. Plant Cell.

[45] Theodoris G, Inada N, Freeling M (2003). Conservation and molecular dissection of ROUGH SHEATH2 and ASYMMETRIC LEAVES1 function in leaf development.. Proc Natl Acad Sci U S A.

[46] Chalfun-Junior A, Franken J, Mes JJ, Marsch-Martinez N, Pereira A (2005). ASYMMETRIC LEAVES2-LIKE1 gene, a member of the AS2/LOB family, controls proximal-distal patterning in Arabidopsis petals.. Plant Mol Biol.

[47] Hanson J, Johannesson H, Engstrom P (2001). Sugar-dependent alterations in cotyledon and leaf development in transgenic plants expressing the HDZhdip gene ATHB13.. Plant Mol Biol.

[48] Wagner TA, Kohorn BD (2001). Wall-associated kinases are expressed throughout plant development and are required for cell expansion.. Plant Cell.

[49] Yang M, Sack FD (1995). The too many mouths and four lips mutations affect stomatal production in Arabidopsis.. Plant Cell.

[50] Berger D, Altmann T (2000). A subtilisin-like serine protease involved in the regulation of stomatal density and distribution in Arabidopsis thaliana.. Genes Dev.

[51] Bergmann DC, Lukowitz W, Somerville CR (2004). Stomatal development and pattern controlled by a MAPKK kinase.. Science.

[52] Shpak ED, McAbee JM, Pillitteri LJ, Torii KU (2005). Stomatal patterning and differentiation by synergistic interactions of receptor kinases.. Science.

[53] Jack T (2004). Molecular and Genetic Mechanisms of Floral Control.. The Plant Cell.

[54] Ziegelhoffer EC, Medrano LJ, Meyerowitz EM (2000). Cloning of the Arabidopsis *WIGGUM* gene identifies a role for farnesylation in meristem development.. Proc Natl Acad Sci U S A.

[55] Fletcher JC (2001). *The ULTRAPETALA* gene controls shoot and floral meristem size in Arabidopsis.. Development.

[56] Running MP, Lavy M, Sternberg H, Galichet A, Gruissem W (2004). Enlarged meristems and delayed growth in *plp* mutants result from lack of CaaX prenyltransferases.. Proc Natl Acad Sci U S A.

[57] Noh YS, Amasino RM (2003). *PIE1*, an ISWI family gene, is required for FLC activation and floral repression in Arabidopsis.. Plant Cell.

[58] Clark SE, Running MP, Meyerowitz EM (1993). *CLAVATA1*, a regulator of meristem and flower development in Arabidopsis.. Development.

[59] Kayes JM, Clark SE (1998). *CLAVATA2*, a regulator of meristem and organ development in Arabidopsis.. Development.

[60] Clark SE, Running MP, Meyerowitz EM (1995). *CLAVATA3* is a specific regulator of shoot and floral meristem development affecting the same processes as *CLAVATA1*.. Development.

[61] Palusa SG, Ali GS, Reddy AS (2007). Alternative splicing of pre-mRNAs of Arabidopsis serine/arginine-rich proteins: regulation by hormones and stresses.. Plant J.

[62] Lopato S, Kalyna M, Dorner S, Kobayashi R, Krainer AR (1999). atSRp30, one of two SF2/ASF-like proteins from Arabidopsis thaliana, regulates splicing of specific plant genes.. Genes Dev.

[63] Lazar G, Goodman HM (2000). The Arabidopsis splicing factor SR1 is regulated by alternative splicing.. Plant Mol Biol.

[64] Kalyna M, Lopato S, Barta A (2003). Ectopic expression of atRSZ33 reveals its function in splicing and causes pleiotropic changes in development.. Mol Biol Cell.

[65] Thompson JD, Gibson TJ, Plewniak F, Jeanmougin F, Higgins DG (1997). The CLUSTAL_X windows interface: flexible strategies for multiple sequence alignment aided by quality analysis tools.. Nucleic Acids Res.

[66] Prasad J, Colwill K, Pawson T, Manley JL (1999). The protein kinase Clk/Sty directly modulates SR protein activity: both hyper-and hypophosphorylation inhibit splicing.. Mol Cell Biol.

[67] Li C, Wong WH (2001). Model-based analysis of oligonucleotide arrays: expression index computation and outlier detection.. Proc Natl Acad Sci U S A.

[68] Benjamini Y, Hochberg Y (1995). Controlling the false discovery rate—A practical and powerful approach to multiple testing.. J R Stat Soc Ser B Methodol.

[69] Reddy VS, Day IS, Thomas T, Reddy AS (2004). KIC, a novel Ca2+ binding protein with one EF-hand motif, interacts with a microtubule motor protein and regulates trichome morphogenesis.. Plant Cell.

[70] He Y, Amasino RM (2005). Role of chromatin modification in flowering-time control.. Trends Plant Sci.

