# Supplementary figures and images for: Regulation of Plant Developmental Processes by a Novel Splicing Factor
Source: PLoS One. 2007 May 30;2(5):e471. doi: 10.1371/journal.pone.0000471 (PMC1868597; doi:10.1371/journal.pone.0000471)

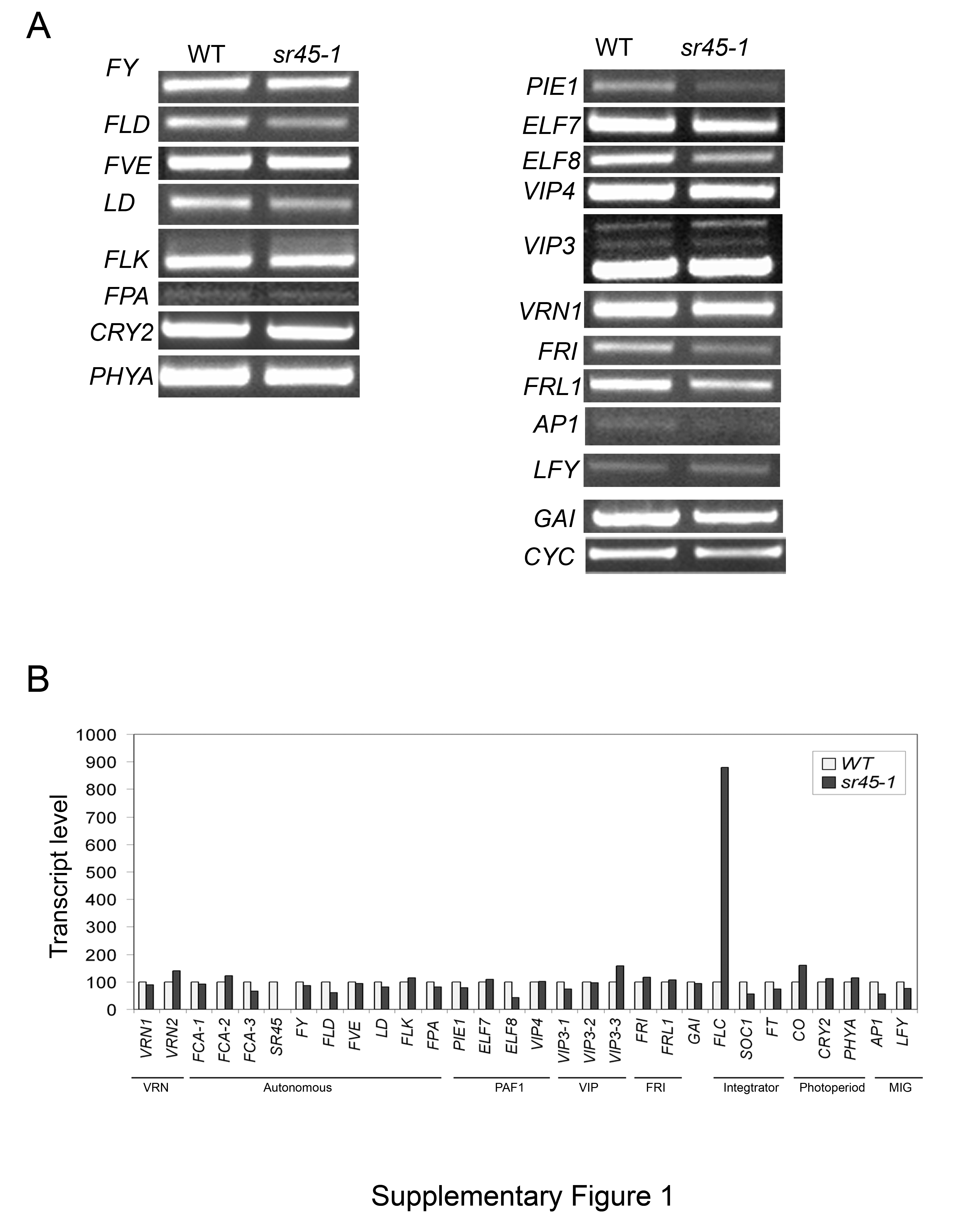

Supplement: Figure S1 — Expression analyses of flowering time genes in WT and sr45-1 plants. (A) RT-PCR was performed with total RNA isolated from two week old plants with gene-specific primers as described in the Experimental procedures. For full name of genes see the legend of Figure S1B below. (B) PCR bands were quantified with NIH Image J software (http://rsb.info.nih.gov/ij/). Each PCR product in WT and sr45-1 was normalized to cyclophilin transcript level in WT and sr45-1, respectively. Data shown are the percent of WT, with WT levels adjusted to 100 percent. VRN, The vernalization pathway; Autonomous, The autonomous flowering pathway; PAF1, RNA polymerase II (Pol II) Associated Factor 1-complex; VIP, vernalization independence; Integrator; Floral pathway integrators; Photoperiod, Photoperiod pathway genes; MIG, meristem identity genes. VRN1,VERNALIZATION 1; VRN2, VERNALIZATION 2; FCA, FCA protein; FY, FY protein; FLD, Flowering Locus D; FVE, FVE protein; LD, LUMINIDEPENDENS; FLK, FLOWERING LATE KH DOMAIN; PIE1, PHOTOPERIOD INDEPENDENT EARLY FLOWERING1; ELF7, EARLY FLOWERING 7; ELF8, EARLY FLOWERING 8; VIP4, VERNALIZATION INDEPENDENCE 4; VIP3, VERNALIZATION INDEPENDENCE 3; FRI, FRIGIDA; FRL1, FRIGIDA-LIKE 1; GAI, GA INSENSITIVE; FLC, FLOWERING LOCUS C; SOC1, SUPPRESSER OF OVEREXPRESSER OF CONSTANS 1; FT, FLOWERING TIME T; CRY2, CRYPTOCHROME2; PHYA, PHYTOCHROME A; AP1, APETELLA 1; LFY, LEAFY; CYC, CYCLOPHILLIN. (1.19 MB TIF) [file pone.0000471.s001.tif]

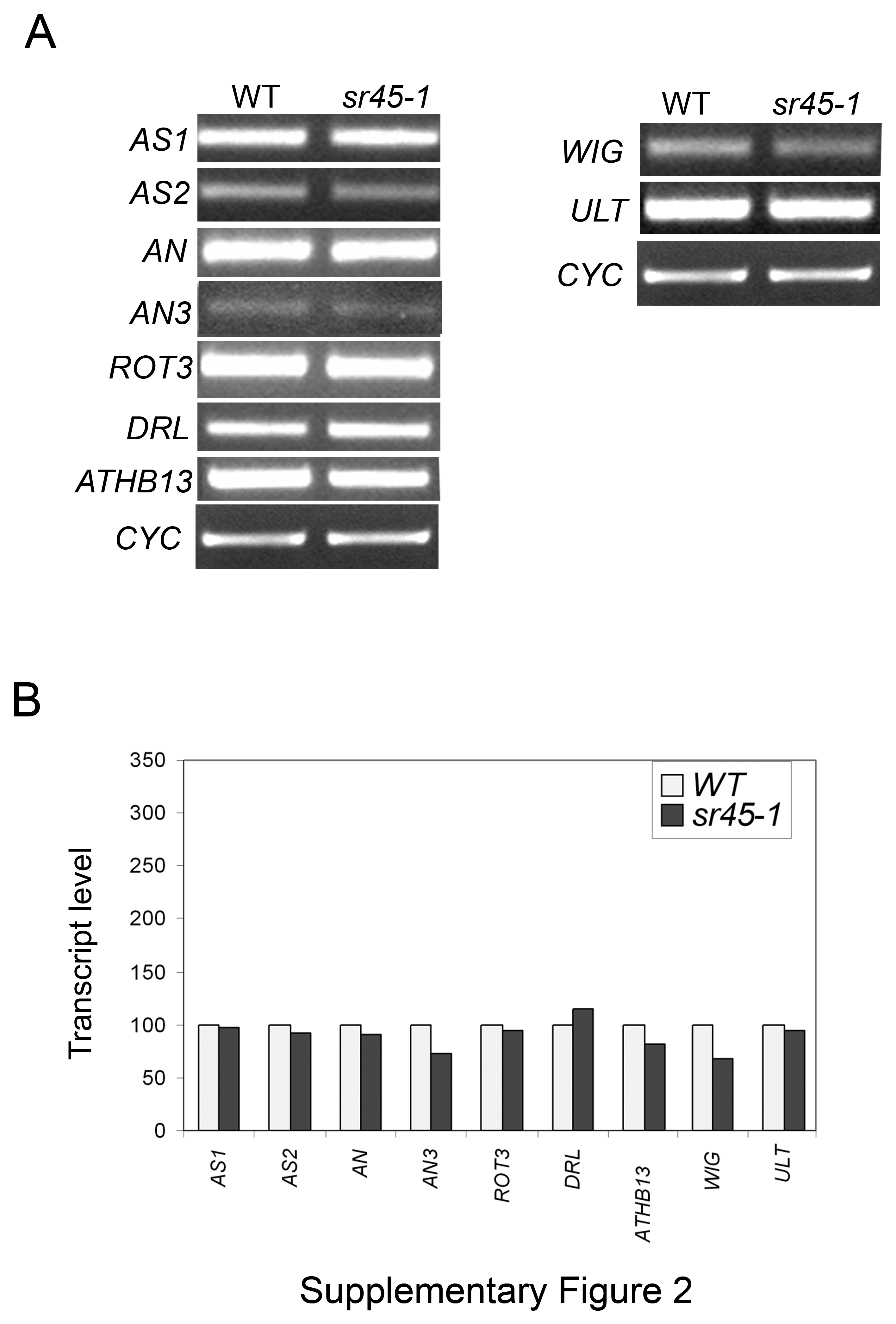

Supplement: Figure S2 — Expression analyses of leaf shape and floral organ number genes in WT and sr45-1 plants. (A) RT-PCR was performed with total RNA isolated from two week old plants with gene-specific primers as described in the Experimental procedures. Left panel consists of RT-PCR of leaf morphology genes; right panel consists of genes affecting petal numbers. AS1, ASYMMETRIC LEAVES1; AS2, ASYMMETRIC LEAVES2; AN, ANGUSTIFOLIA; AN3, ANGUSTIFOLIA3; ROT3, ROTUNDIFOLIA3; DRL, DEFORMED ROOTS AND LEAVES 1; ATHB13, HOMEODOMAIN LEUCINE-ZIPPER PROTEIN ATHB13; WIG, WIGGUM; ULT, ULTRAPETALLA; CYC, CYCLOPHILLIN. (B) PCR bands were quantified with NIH Image J software (http://rsb.info.nih.gov/ij/). Each PCR product in WT and sr45-1 was normalized to cyclophilin transcript level in WT and sr45-1, respectively. Data shown are the percent of WT, with WT levels adjusted to 100 percent. (0.74 MB TIF) [file pone.0000471.s002.tif]
