# Supplementary material for: Regulation of Plant Developmental Processes by a Novel Splicing Factor
Source: PLoS One. 2007 May 30;2(5):e471. doi: 10.1371/journal.pone.0000471 (PMC1868597; doi:10.1371/journal.pone.0000471)
Supplement: Table S1 — Summary of Microarray Analyses of Flowering Time Genes in sr45-1 and WT Arabidopsis. Gene expression data of sr45-1 and WT consisting of three biological replicates were statistically analyzed as described in Materials and Methods. (0.11 MB DOC) [file pone.0000471.s003.doc]

Supplementary Table 1: Summary of Microarray Analyses of Flowering Time Genes in *sr45-1* and WT Arabidopsis.

| Gene name | Affymetrix Probe set ID | (*sr45-1*/WT) Signal Log ratio^1^ | Moderated *p*-value^2^ | FDR corrected *p*-value^3^ | AGI identifier | Effect on flowering | Gene Description |
| --- | --- | --- | --- | --- | --- | --- | --- |
| **Autonomous pathway** |  |  |  |  |  |  |  |
| *FLC* | 250476_at | 2.79 | 4.63e-07 | 0.0067976 | AT5G10140 | delays | *FLOWERING LOCUS C* |
| *FCA* | 245489_at | 0.04 | 0.736709411 | 0.8861321 | AT4G16280 | promotes | *FCA protein* |
| *FY* | 245848_at | -0.06 | 0.635851773 | 0.8349682 | AT5G13480 | promotes | *FY protein* |
| *FLD* | 258944_at | 0.07 | 0.598440446 | 0.8125689 | AT3G10390 | promotes | *Flowering Locus D* |
| *FVE* | 265946_s_at | -0.08 | 0.475949504 | 0.7400534 | AT2G19520 | promotes | *FVE protein* |
| *LD* | 255444_at | -0.40 | 0.012538182 | 0.1643808 | AT4G02560 | promotes | *LUMINIDEPENDENS* |
| *FLK* | 258790_at | -0.03 | 0.838807406 | 0.9366063 | AT3G04610 | promotes | *FLOWERING LOCUS KH DOMAIN* |
| **Vernalization pathway** |  |  |  |  |  |  |  |
| *VRN1* | 256944_at | -0.17 | 0.144826185 | 0.4518546 | AT3G18990 | promotes | *VERNALIZATION 1* |
| *VRN2* | 245280_at | -0.12 | 0.288862082 | 0.5989254 | AT4G16845 | promotes | *VERNALIZATION 2* |
| **PAF1-Complex** |  |  |  |  |  |  |  |
| *PIE1* | 257688_at | -0.05 | 0.660276487 | 0.8463348 | AT3G12810 | delays | *PHOTOPERIOD INDEPENDENT EARLY FLOWERING 1* |
| *ELF7* | 261347_at | -0.14 | 0.267409235 | 0.5784244 | AT1G79730 | delays | *EARLY FLOWERING 7* |
| *ELF8* | 265522_at | -0.20 | 0.080033871 | 0.3520652 | AT2G06210 | delays | *EARLY FLOWERING 8* |
| *VIP4* | 247565_at | -0.13 | 0.214785946 | 0.529382 | AT5G61150 | delays | *VERNALIZATION INDEPENDENCE 4* |
|  |  |  |  |  |  |  |  |
| *FRI* | 255634_at | -0.28 | 0.029761 | 0.229611 | AT4G00650 | delays | *FRIGIDA* |
| *FRL1* | 250113_at | 0.00 | 0.9703747 | 0.989513 | AT5G16320 | delays | *FRIGIDA-LIKE 1* |
| *VIP3* | 253645_at | 0.04 | 0.6968997 | 0.866138 | AT4G29830 | delays | *VERNALIZATION INDEPENDENCE 3* |
| **Photoperiod pathway** |  |  |  |  |  |  |  |
| *CO* | 260488_at | -0.14 | 0.2789522 | 0.590634 | AT5G15840 | promotes | *CONSTANS* |
| *PHYA* | 264508_at | -0.08 | 0.5533104 | 0.785271 | ATGg09570 | promotes | *PHYTOCHROME A* |
| *CRY2* | 263669_at | -0.18 | 0.1210393 | 0.419326 | AT1G04400 | promotes | *CRYPTOCHROME* |
| *GI* | 264211_at | -0.19 | 0.2148034 | 0.529382 | AT1G22770 | promotes | *GIGANTEA* |
| **Gibberellins pathway** |  |  |  |  |  |  |  |
| *GAI* | 262850_at | -0.02 | 0.8778261 | 0.953067 | AT1G14920 | promotes | *GA INSENSITIVE* |
| **Floral integrator** |  |  |  |  |  |  |  |
| *SOC1/AGL20* | 267509_at | -1.14 | 0.001874 | 0.078479 | AT2G45660 | promotes | *SUPPRESSER OF OVEREXPRESSER OF CONSTANS 1* |
| *LFY* | 247490_at | 0.24 | 0.0846604 | NA | AT5G61850 | promotes | *LEAFY* |
| *FT* | 264638_at | -0.08 | 0.4838257 | 0.744665 | AT3G04610 | promotes | *FLOWERING TIME T* |
| *AP1* | 259372_at | 0.07 | 0.586041 | NA | AT1G69120 | promotes | *APETELLA 1* |

^1^Average log ratios of gene expressions in *sr45-1* as compared to WT; negative numbers indicate repression, whereas, positive numbers indicate induction in *sr45-1*. ^2^Moderated *p*-values are the probabilities of *t*-test statistics. ^3^False discovery rate (FDR) corrected *p*-values are *p*-values that were adjusted for multiple testing according Benjamini-Hochberg method (see Materials and Methods for details).
