# Supplementary material for: Regulation of Plant Developmental Processes by a Novel Splicing Factor
Source: PLoS One. 2007 May 30;2(5):e471. doi: 10.1371/journal.pone.0000471 (PMC1868597; doi:10.1371/journal.pone.0000471)
Supplement: Table S3 — Sequences of gene-specific primers of Arabidopsis SR, flowering pathway and leaf morphology genes. (0.08 MB DOC) [file pone.0000471.s005.doc]

Supplementary Table 3. Sequences of gene-specific primers of Arabidopsis SR, flowering pathway and leaf morphology

genes.

| Name | Gene ID | Forward primer | Reverse primer |
| --- | --- | --- | --- |
| *SRp30 (ASF/SF2-like)* | At1g09140 | CGCAAGTGTGAGGTTGAAGA | ATGCAGCCGAGACAGAGTTT |
| *SR1/SRp34 (ASF/SF2-like* | At1g02840 | TCGACGACCAACAGAATGAG | GCTAGGGCTCTTGCTTCCTT |
| *SRp34a (ASF/SF2-like)* | At3g49430 | TTGGCTTCAGACCAAATCTTC | TTCTTTTGGCCATTTTCACC |
| *SRp34b (ASF/SF2-like)* | At4g02430 | GGCGATATCCGTGAAAGAGA | TCTTCCAACAGACCCAGCTT |
| *RSp31* | At3g61860 | GGAACGGTTGTTCGACAAGT | GGACTTGGACGCCTACGATA |
| *RSp31a* | At2g46610 | AGCAAGTTCGGGAGAGTGAA | GCCCAACAACATCTTCAACC |
| *RSp40/SRp35* | At4g25500 | ACTACGCCTGCCAAAATCAT | CACCATCATACCCACCATCA |
| *RSp41* | At5g52040 | GAGAGCCTCGAAGAAAGCAA | GCGATTTCGAATGGAGTCAT |
| *SRZ21/RSZ21 (9G8-like)* | At1g23860 | TGCAACATGACGAGGGTTTA | AAAAGGCGCCACAGAGTAGA |
| *SRZ22/RSZ22 (9G8-like)* | At4g31580 | CCCTTGAGTGCTTTCAGCTC | GAAACGCTTAGGCATTTAGCA |
| *RSZ22a (9G8-like)* | At2g24590 | AAGAGCCAAAGCCGTTTCTT | TGCATAGGTTTTTAGCAGAGCTT |
| *RSZ32* | At3g53500 | GTATCATCCGCGGTTCACTT | TGTCCTCCACGCTTTTCTCT |
| *RSZ33* | At2g37340 | GCAGTGCTCTCCTTCAATCC | ACATGCTACAATGCCTGCAA |
| *SC35* | At5g64200 | CCTTCCGTACGACTGCTGAT | TCAACATGGTTGTGCCATCT |
| *SR33/SCL33 (SC35-like)* | At1g55310 | CTCCGTCGTTCCTCACCACCG | GTTCCCCACATGTTCCATAG |
| *SCL30 (SC35-like)* | At3g55460 | CTCCTCGACGTGGATATGGT | ACCTTCATAGCCAGGGGAGT |
| *SCL30a (SC35-like)* | At3g13570 | TTCCCCTGTGTTTTTCTTCG | CTTTGGCTCCTTGCTTGTTC |
| *SCL28 (SC35-like)* | At5g18810 | TCGTTTTCCGACAGGGTTAG | TTGCCTACTTCAAAGCCAAAA |
| *SR45* | At1g16610 | TGGCGAAACCAAGTCGTGG | TTAAGTTTTACGAGGTGGAG |
| *VRN2* | At4g16845 | ATGTGTAGGCAGAATTGTCGCG | TTACTTGTCTCTGCTGTTATTG |
| *FLC* | At5g10140 | ATGGGAAGAAAAAAACTAGAAATCAA | CTAATTAAGTAGTGGGAGAGTCAC |
| *SOC1* | At2g45660 | ATGGTGAGGGGCAAAACTCAGATGAA | TTCATGAGATCCCCACTTTTCAGAGA |
| *FT* | At3g04610 | ATGTCTATAAATATAAGAGACCCTC | CTAAAGTCTTCTTCCTCCGCAGCCAC |
| *FCAEXON13F* | At4g16280 | CAAAATCGGGCAGCTGGCCAG |  |
| *FCAEXON14R* | At4g16280 |  | CTTGCTTTCACCCGTTAGACCA |
| *CO* | At5g15840 | CACACCATCAAACTTACTACATCTG | CTGAAAATTCTGTTGGTTATGGCAC |
| *FY* | At5g13480 | ATGGCAATTGGCTTTTAACG | GCCACTGTTTGGTTGTCCTT |
| *FLD* | At3g10390 | CTGACGCAGTGACTCGTGTT | TTGCTGCTGGTTGAAATGAG |
| *FVE* | At2g19520 | ATGGCAATTGGCTTTTAACG | GCCACTGTTTGGTTGTCCTT |
| *LD* | At4g02560 | GTCAATGGACCTTTGGCTGT | ATTGCATTGAACCAACACGA |
| *FLK* | At3g04610 | ATTCCGTATGCTGGTTCCTG | GAATCTGCATTTGCTGCGTA |
| *VRN1* | At3g18990 | CGTCTGAGGGTCCCAGATAA | ATCGAACAGGCCATTGTTTC |
| *PIE1* | At3g12810 | TTGGCTGTCGAAGAGGAACT | ATTCTGCAGGGGTGTACCAG |
| *ELF7* | At1g79730 | AACCAACCACCTTCATCTGC | CTCATCCAAGGAAGGAACCA |
| *ELF8* | At2g06210 | CGCGAGGGAGTACTTCAAAC | TCCTTTAAGCTCCCCCAACT |
| *VIP4* | At5g61150 | CGGAGAAGCTGAAGCTGAGT | CCACCTTACAAACCGAGCAT |
| *FRI* | At4g00650 | TCTGAACAGCGACGAAGAGA | GAAACTGTGACCGCTTCAAA |
| *FRL1* | At5g16320 | GCTTCTGATGCAGGGAAATC | TAGTCCGCTTTTTCCCCTTT |
| *VIP3* | At4g29830 | AACTCGCAGGTCTGAAATCG | TGGTCATTGTGGTTGCTCAT |
| *PHYA* | At1g09570 | TGTGCTGAACAATGCTGTGA | CCTCAGGGAAGCTGAAACAG |
| *CRY2* | At1g04400 | GTGGAACGTGGGATCTCTGT | ATATTTGGCGCCTTGTAACG |
| *GI* | At1g22770 | CAGCAGAAGCAACCATTGAA | TCACAGGCAAGAGCACAAAC |
| *AP1* | At1g69120 | CGCCGAAAGACAGCTTATTG | CAGCCAAGGTTGCAGTTGTA |
| *FPA* | At2g43410 | ATTCTGGCAACCGTATCGTC | GCGCTCCTAAACTCCACAAG |
| *AS1* | At2g37630 | AGACAGTTCGGTCCGAGAGA | CCACTGTGGAAGGCGATAAT |
| *AS2* | At1g65620 | TGTGTATTCGCGCCCTATTT | AGTACGGCGACCATCATCTC |
| *AN* | At1g01510 | TACTCTCGCGACACGCTCTA | CCTGTTGCCTACTGGTGGAT |
| *ROT3* | At4g36380 | ACGGGAAAGTGTTCAAAACG | TCCTTGAGAGCTTTCCTCCA |
| *DRL* | At1g13870 | GTGGGCAACCTTGTAGTGGT | TAACGGTGGACCGCTTAGAC |
| *ATHB13* | At1g69780 | CTCCATGGATTTGCTTCGTT | ATGGTCATCCATTGCACAGA |
| *WIGGUM* | At5g40280 | ACCCAGGGCCTAGGAGATTA | GTTTCCTCGGCTTGTCTCTG |
| *ULTRAPETALA* | At4g28190 | ATGTTGGTGGCGATTACGTT | ACCTTCCTGCTCCCTCTCTC |
| *AN3* | At5g28640 | GCTGGTTACTACCCCAGCAA | TCCCCTGAACTTCCTCCTCT |
